# (3,6-Dimeth­oxy­naphthalen-2-yl)(phen­yl)methanone

**DOI:** 10.1107/S1600536811005630

**Published:** 2011-02-19

**Authors:** Yuichi Kato, Ryo Takeuchi, Toyokazu Muto, Akiko Okamoto, Noriyuki Yonezawa

**Affiliations:** a Department of Organic and Polymer Materials Chemistry, Tokyo University of Agriculture & Technology, 2-24-16 Naka-machi, Koganei, Tokyo 184-8588, Japan

## Abstract

In the title compound, C_19_H_16_O_3_, the dihedral angle between the naphthalene ring system and the phenyl ring is 68.32 (5)°. The bridging carbonyl C—C(=O)—C plane makes a dihedral angle of 54.32 (5)° with the naphthalene ring system and 21.45 (6)° with the phenyl ring. An inter­molecular C—H⋯O hydrogen bond exists between the H atom of one meth­oxy group and the O atom of the second meth­oxy group in an adjacent mol­ecule. The crystal packing is additionally stabilized by a weak C—H⋯O inter­molecular inter­action between an H atom of the naphthalene ring and the O atom of the carbonyl group.

## Related literature

For electrophilic aromatic substitution of naphthalene deriv­atives affording *peri-*aroylated compounds regioselectively, see: Okamoto & Yonezawa (2009 ▶). For the structures of closely related compounds, see: Kataoka *et al.* (2010 ▶); Kato *et al.* (2010 ▶); Muto *et al.* (2010 ▶); Nakaema, Okamoto *et al.* (2008 ▶); Nakaema, Watanabe *et al.* (2008 ▶); Nishijima *et al.* (2010 ▶); Watanabe *et al.* (2010 ▶).
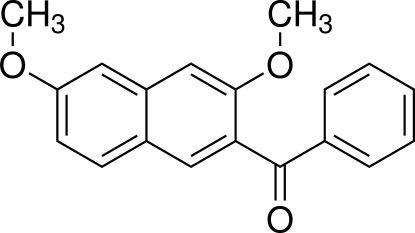


## Experimental

### 

#### Crystal data

C_19_H_16_O_3_
*M* *_r_* = 292.32
Monoclinic, 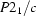

*a* = 8.7186 (2) Å
*b* = 20.4650 (4) Å
*c* = 8.5675 (2) Å
β = 102.475 (1)°
*V* = 1492.57 (6) Å^3^
*Z* = 4
Cu *K*α radiation
μ = 0.71 mm^−1^
*T* = 193 K
0.60 × 0.50 × 0.20 mm

#### Data collection

Rigaku R-AXIS RAPID diffractometer
Absorption correction: numerical (*NUMABS*; Higashi, 1999 ▶) *T*_min_ = 0.677, *T*_max_ = 0.872
26682 measured reflections
2735 independent reflections
2509 reflections with *I* > 2σ(*I*)
*R*_int_ = 0.044

#### Refinement

*R*[*F* ^2^ > 2σ(*F* ^2^)] = 0.034
*wR*(*F* ^2^) = 0.098
*S* = 1.06
2735 reflections
202 parameters
H-atom parameters constrained
Δρ_max_ = 0.23 e Å^−3^
Δρ_min_ = −0.17 e Å^−3^

### 

Data collection: *PROCESS-AUTO* (Rigaku, 1998 ▶); cell refinement: *PROCESS-AUTO*; data reduction: *CrystalStructure* (Rigaku, 2010 ▶); program(s) used to solve structure: *Il Milione* (Burla *et al.*, 2007 ▶); program(s) used to refine structure: *SHELXL97* (Sheldrick, 2008 ▶); molecular graphics: *ORTEPIII* (Burnett & Johnson, 1996 ▶); software used to prepare material for publication: *SHELXL97*.

## Supplementary Material

Crystal structure: contains datablocks I, global. DOI: 10.1107/S1600536811005630/fk2037sup1.cif

Structure factors: contains datablocks I. DOI: 10.1107/S1600536811005630/fk2037Isup2.hkl

Additional supplementary materials:  crystallographic information; 3D view; checkCIF report

## Figures and Tables

**Table 1:** Hydrogen-bond geometry (Å, °)

*D*—H⋯*A*	*D*—H	H⋯*A*	*D*⋯*A*	*D*—H⋯*A*
C4—H4⋯O1^i^	0.95	2.58	3.4439 (13)	151
C18—H18*B*⋯O3^ii^	0.98	2.42	3.3742 (15)	164

Symmetry codes: (i) 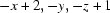; (ii) 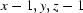.

**Fig. 1.**
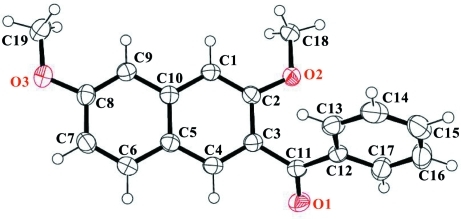
Molecular structure with displacement ellipsoids at 50% probability level for non-H atoms.

**Fig. 2.**
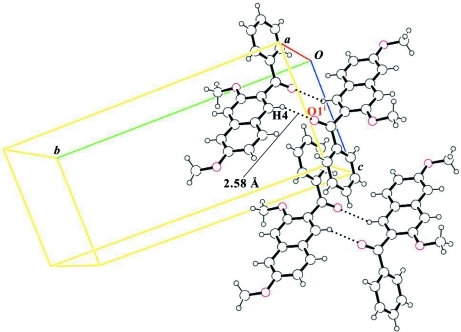
The C4—H4···O1 intermolecular interaction [symmetry code: (i) – x + 2, – y, – z + 1].

**Fig. 3.**
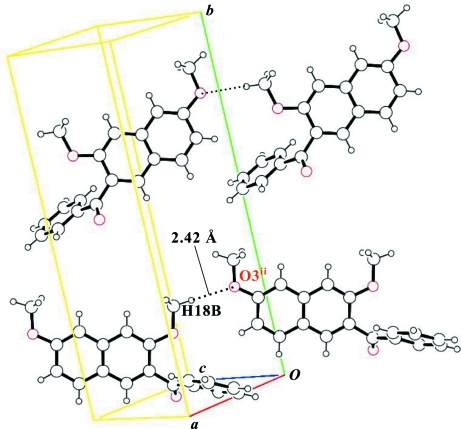
The C18—H18B···O3 intermolecular interaction [symmetry code: (ii) x – 1, y, z – 1].

## supplementary crystallographic information

### Comment

In the course of our study on selective electrophilic aromatic aroylation of 2,7-dimethoxynaphthalene, *peri-*aroylnaphthalene compounds have proved to be formed regioselectively with the aid of suitable acidic mediator (Okamoto & Yonezawa, 2009). Recently, we have reported the structures of 1,8-diaroyl-2,7-dimethoxynaphthalenes such as 1,8-bis(4-methylbenzoyl)-2,7-dimethoxynaphthalene (Muto *et al.*, 2010) and 1,8-bis(4-aminobenzoyl)-2,7-dimethoxynaphthalene (Nishijima *et al.*, 2010). The aroyl groups at the 1,8-positions of the naphthalene rings in these compounds are bonded in a nearly perpendicular manner but the benzene rings of the aroyl groups tilt slightly toward the *exo* sides of the naphthalene rings. Such 1-aroylnaphthalene homologues as (2,7-dimethoxynaphthalen-1-yl)(3-nitrophenyl)methanone (Kataoka *et al.*, 2010) are also revealed to have essentially the same non-coplanar structure as observed for 1,8-diaroylated naphthalenes. Furthermore, we reported the crystal structure analysis of the corresponding *β*-isomers of 3-aroyl-2,7-dimethoxynaphthalenes such as 2-(4-chlorobenzoyl)-3,6-dimethoxynaphthalene (Nakaema, Okamoto *et al.*, 2008) and (4-fluorophenyl) (3,6-dimethoxy-2-naphthyl)methanone (Watanabe *et al.*, 2010). In the 3-aroylated naphthalenes, which are generally regarded to be thermodynamically more stable than the corresponding 1-positioned isomeric molecules, the aroyl groups are connected to the naphthalene rings in a moderately twisted fashion. On the other hand, there are several unique structural features in the benzoylated naphthalene homologues, 1-benzoyl-2,7-dimethoxynaphthalene (Kato *et al.*, 2010) and 1,8-dibenzoyl-2,7-dimethoxynaphthalene (Nakaema, Watanabe *et al.*, 2008). 1-Benzoyl-2,7-dimethoxynaphthalene contains three independent conformers and each of them forms a columnar structure, respectively. As a part of our ongoing study on the synthesis and structure of these homologous molecules, the crystal structure analysis of the title compound, a 3-monoaroylnaphthalene, is discussed in this article.

The molecular structure of the title molecule is displayed in Fig. 1. The benzene group is bonded to the naphthalene ring with a non-coplanar configuration. The dihedral angle between the best planes of the benzene ring (C12—C17) and the naphthalene ring (C1—C10) is 68.32 (5)°. The bridging carbonyl plane (O1—C3—C11—C12) makes a relatively large dihedral angle of 54.32 (5)° with the naphthalene ring (C1—C10) [C2—C3—C11—O1 torsion angle = -125.86 (12)°], whereas it makes a rather small dihedral angle of 21.45 (6)° with benzene ring (C12—C17) [O1—C11—C12—C13 torsion angle = -156.47 (11)°].

The crystal packing exhibits a weak C—H···O intermolecular interaction between the oxygen atom of the carbonyl group and the hydrogen atom of the naphthalene ring (Table 1, Fig. 2). The packing is additionally stabilized by a C—H···O hydrogen bond between the hydrogen of the 2-methoxy group, which is situated adjacent to the benzoyl group, and the ethereal oxygen atom of the 7-methoxy group in the neighboring molecule (Table 1, Fig. 3).

### Experimental

A mixture of 2,7-dimethoxynaphthalene (3.74 g, 19.9 mmol), FeCl_3_ (4.95 g, 37.1 mmol), trichloromethylbenzene (2.9 ml, 20 mmol) and dichloromethane (50 ml) was stirred at 293 K for 6 h, and the reaction mixture was poured into ice-cooled water followed by extraction with CHCl_3_ (30 ml × 3). The combined extracts were washed with 2 *M* aqueous NaOH followed by washing with brine. The organic layer thus obtained was dried over anhydrous MgSO_4_. The solvent was removed under reduced pressure to give cakes (yield 72%). The crude product was purified by flush silica gel chromatography (CHCl_3_). Colorless platelet single crystals suitable for X-ray diffraction were obtained by crystallization from hexane and chloroform (yield 21%).

Spectroscopic Data:

^1^H NMR δ (400 MHz, CDCl_3_); 3.83 (3*H*, s), 3.95 (3*H*, s), 7.05 (1*H*, dd, *J* = 2.4, 9.2 Hz), 7.11 (1*H*, d, *J* = 2.4 Hz), 7.14 (1*H*, s), 7.44 (2*H*, t, *J* = 8.0 Hz), 7.56 (1*H*, t, *J* = 7.6 Hz), 7.69 (1*H*, d, *J* = 9.2 Hz), 7.78 (1*H*, s), 7.83–7.85 (2*H*, m) p.p.m..

^13^C NMR δ (75 MHz, CDCl_3_); 55.34, 55.54, 105.00, 105.38, 117.02, 123.15, 127.89, 128.18, 129.93, 130.01, 130.07, 132.87, 137.11, 138.06, 155.83, 159.30, 196.02 p.p.m..

IR (KBr): 1627 (C═O), 1580, 1502 (Ar, naphthalene), 1213 cm^-1^.

HRMS (*m/z*): [*M* + H]^+^ Calcd for C_19_H_17_O_3_, 293.1178; found, 293.1203.

m.p. = 438.7–441.5 K.

### Refinement

H atom positions were derived from geometrical considerations and were subsequently refined as riding atoms, with C—H = 0.95 (aromatic) and 0.98 (methyl) Å, and with *U*_iso_(H) = 1.2*U*_eq_(C) or 1.5(methyl).

### Crystal data

**Table:**

C_19_H_16_O_3_	*F*(000) = 616
*M**_r_* = 292.32	*D*_x_ = 1.301 Mg m^−^^3^
Monoclinic, *P*2_1_/*c*	Melting point = 438.7–441.5 K
Hall symbol: -P 2ybc	Cu *K*α radiation, λ = 1.54187 Å
*a* = 8.7186 (2) Å	Cell parameters from 16018 reflections
*b* = 20.4650 (4) Å	θ = 4.3–68.2°
*c* = 8.5675 (2) Å	µ = 0.71 mm^−^^1^
β = 102.475 (1)°	*T* = 193 K
*V* = 1492.57 (6) Å^3^	Platelet, colorless
*Z* = 4	0.60 × 0.50 × 0.20 mm

### Data collection

**Table:**

Rigaku R-AXIS RAPID diffractometer	2735 independent reflections
Radiation source: rotating anode	2509 reflections with *I* > 2σ(*I*)
graphite	*R*_int_ = 0.044
Detector resolution: 10.000 pixels mm^-1^	θ_max_ = 68.2°, θ_min_ = 4.3°
ω scans	*h* = −10→10
Absorption correction: numerical (*NUMABS*; Higashi, 1999)	*k* = −24→24
*T*_min_ = 0.677, *T*_max_ = 0.872	*l* = −10→10
26682 measured reflections	

### Refinement

**Table:**

Refinement on *F*^2^	Secondary atom site location: difference Fourier map
Least-squares matrix: full	Hydrogen site location: inferred from neighbouring sites
*R*[*F*^2^ > 2σ(*F*^2^)] = 0.034	H-atom parameters constrained
*wR*(*F*^2^) = 0.098	*w* = 1/[σ^2^(*F*_o_^2^) + (0.0584*P*)^2^ + 0.2227*P*] where *P* = (*F*_o_^2^ + 2*F*_c_^2^)/3
*S* = 1.06	(Δ/σ)_max_ = 0.001
2735 reflections	Δρ_max_ = 0.23 e Å^−^^3^
202 parameters	Δρ_min_ = −0.17 e Å^−^^3^
0 restraints	Extinction correction: *SHELXL97* (Sheldrick, 2008), Fc^*^=kFc[1+0.001xFc^2^λ^3^/sin(2θ)]^-1/4^
Primary atom site location: structure-invariant direct methods	Extinction coefficient: 0.0131 (8)

### Special details

**Table:**

Geometry. All e.s.d.'s (except the e.s.d. in the dihedral angle between two l.s. planes) are estimated using the full covariance matrix. The cell e.s.d.'s are taken into account individually in the estimation of e.s.d.'s in distances, angles and torsion angles; correlations between e.s.d.'s in cell parameters are only used when they are defined by crystal symmetry. An approximate (isotropic) treatment of cell e.s.d.'s is used for estimating e.s.d.'s involving l.s. planes.
Refinement. Refinement of *F*^2^ against ALL reflections. The weighted *R*-factor *wR* and goodness of fit *S* are based on *F*^2^, conventional *R*-factors *R* are based on *F*, with *F* set to zero for negative *F*^2^. The threshold expression of *F*^2^ > σ(*F*^2^) is used only for calculating *R*-factors(gt) *etc*. and is not relevant to the choice of reflections for refinement. *R*-factors based on *F*^2^ are statistically about twice as large as those based on *F*, and *R*- factors based on ALL data will be even larger.

### Fractional atomic coordinates and isotropic or equivalent isotropic displacement parameters (Å^2^)

**Table:**

	*x*	*y*	*z*	*U*_iso_*/*U*_eq_	
O1	0.78878 (10)	0.03272 (4)	0.31564 (10)	0.0421 (2)	
O2	0.82137 (9)	0.20198 (4)	0.16422 (9)	0.0365 (2)	
O3	1.49637 (9)	0.28351 (4)	0.80270 (10)	0.0420 (2)	
C1	1.04784 (12)	0.23386 (5)	0.36717 (13)	0.0299 (2)	
H1	1.0346	0.2786	0.3379	0.036*	
C2	0.94725 (12)	0.18810 (5)	0.28459 (13)	0.0297 (2)	
C3	0.96544 (12)	0.12068 (5)	0.32677 (12)	0.0301 (2)	
C4	1.08301 (13)	0.10241 (5)	0.45329 (12)	0.0314 (3)	
H4	1.0931	0.0577	0.4834	0.038*	
C5	1.18928 (12)	0.14824 (5)	0.53981 (12)	0.0307 (3)	
C6	1.31320 (13)	0.13009 (5)	0.66941 (13)	0.0361 (3)	
H6	1.3272	0.0854	0.6992	0.043*	
C7	1.41212 (13)	0.17582 (6)	0.75144 (14)	0.0378 (3)	
H7	1.4948	0.1629	0.8374	0.045*	
C8	1.39183 (12)	0.24263 (6)	0.70863 (13)	0.0335 (3)	
C9	1.27567 (12)	0.26199 (5)	0.58315 (13)	0.0314 (3)	
H9	1.2648	0.3069	0.5544	0.038*	
C10	1.17134 (12)	0.21514 (5)	0.49575 (12)	0.0288 (2)	
C11	0.85594 (12)	0.06982 (5)	0.24066 (13)	0.0312 (2)	
C12	0.83398 (13)	0.06328 (5)	0.06375 (13)	0.0320 (3)	
C13	0.94512 (15)	0.08664 (5)	−0.01647 (14)	0.0386 (3)	
H13	1.0356	0.1089	0.0408	0.046*	
C14	0.92417 (18)	0.07753 (6)	−0.18018 (15)	0.0495 (3)	
H14	1.0017	0.0925	−0.2343	0.059*	
C15	0.7911 (2)	0.04677 (6)	−0.26460 (16)	0.0550 (4)	
H15	0.7757	0.0418	−0.3772	0.066*	
C16	0.67992 (18)	0.02314 (7)	−0.18521 (16)	0.0546 (4)	
H16	0.5884	0.0018	−0.2432	0.066*	
C17	0.70235 (15)	0.03068 (6)	−0.02102 (15)	0.0436 (3)	
H17	0.6274	0.0135	0.0337	0.052*	
C18	0.78092 (13)	0.26937 (5)	0.13734 (15)	0.0371 (3)	
H18A	0.7644	0.2890	0.2367	0.044*	
H18B	0.6843	0.2730	0.0545	0.044*	
H18C	0.8663	0.2923	0.1024	0.044*	
C19	1.47866 (16)	0.35166 (6)	0.77225 (16)	0.0468 (3)	
H19A	1.4949	0.3611	0.6648	0.056*	
H19B	1.5563	0.3757	0.8514	0.056*	
H19C	1.3727	0.3652	0.7795	0.056*	

### Atomic displacement parameters (Å^2^)

**Table:**

	*U*^11^	*U*^22^	*U*^33^	*U*^12^	*U*^13^	*U*^23^
O1	0.0504 (5)	0.0376 (4)	0.0393 (5)	−0.0111 (4)	0.0122 (4)	0.0032 (3)
O2	0.0385 (4)	0.0293 (4)	0.0366 (4)	0.0020 (3)	−0.0034 (3)	−0.0004 (3)
O3	0.0390 (5)	0.0418 (5)	0.0411 (5)	−0.0081 (3)	−0.0008 (4)	−0.0024 (4)
C1	0.0344 (5)	0.0250 (5)	0.0309 (6)	0.0011 (4)	0.0086 (4)	0.0016 (4)
C2	0.0317 (5)	0.0303 (5)	0.0273 (5)	0.0022 (4)	0.0067 (4)	0.0012 (4)
C3	0.0344 (5)	0.0282 (5)	0.0291 (5)	−0.0002 (4)	0.0095 (4)	−0.0009 (4)
C4	0.0376 (6)	0.0267 (5)	0.0308 (6)	0.0017 (4)	0.0096 (5)	0.0024 (4)
C5	0.0327 (5)	0.0311 (5)	0.0293 (5)	0.0015 (4)	0.0091 (4)	0.0016 (4)
C6	0.0387 (6)	0.0333 (6)	0.0353 (6)	0.0025 (4)	0.0053 (5)	0.0056 (4)
C7	0.0348 (6)	0.0421 (6)	0.0341 (6)	0.0019 (5)	0.0022 (5)	0.0048 (5)
C8	0.0305 (5)	0.0392 (6)	0.0314 (6)	−0.0038 (4)	0.0079 (4)	−0.0028 (4)
C9	0.0329 (5)	0.0298 (5)	0.0324 (6)	−0.0009 (4)	0.0093 (4)	0.0000 (4)
C10	0.0298 (5)	0.0305 (5)	0.0278 (5)	0.0005 (4)	0.0099 (4)	0.0000 (4)
C11	0.0330 (5)	0.0262 (5)	0.0345 (6)	0.0018 (4)	0.0076 (4)	0.0019 (4)
C12	0.0375 (6)	0.0243 (5)	0.0330 (6)	0.0023 (4)	0.0053 (4)	−0.0010 (4)
C13	0.0491 (7)	0.0298 (5)	0.0386 (6)	−0.0008 (5)	0.0135 (5)	−0.0006 (5)
C14	0.0766 (9)	0.0365 (6)	0.0407 (7)	0.0071 (6)	0.0247 (7)	0.0016 (5)
C15	0.0892 (11)	0.0407 (7)	0.0322 (7)	0.0188 (7)	0.0062 (7)	−0.0044 (5)
C16	0.0614 (8)	0.0492 (8)	0.0447 (8)	0.0046 (6)	−0.0076 (6)	−0.0125 (6)
C17	0.0428 (6)	0.0416 (6)	0.0438 (7)	−0.0027 (5)	0.0034 (5)	−0.0060 (5)
C18	0.0373 (6)	0.0318 (6)	0.0390 (6)	0.0040 (4)	0.0014 (5)	0.0045 (4)
C19	0.0488 (7)	0.0413 (7)	0.0480 (7)	−0.0118 (5)	0.0053 (6)	−0.0059 (5)

### Geometric parameters (Å, °)

**Table:**

O1—C11	1.2224 (13)	C9—C10	1.4188 (15)
O2—C2	1.3637 (13)	C9—H9	0.9500
O2—C18	1.4299 (13)	C11—C12	1.4921 (15)
O3—C8	1.3649 (13)	C12—C13	1.3884 (16)
O3—C19	1.4211 (15)	C12—C17	1.3891 (16)
C1—C2	1.3706 (15)	C13—C14	1.3873 (17)
C1—C10	1.4168 (15)	C13—H13	0.9500
C1—H1	0.9500	C14—C15	1.379 (2)
C2—C3	1.4265 (14)	C14—H14	0.9500
C3—C4	1.3731 (15)	C15—C16	1.386 (2)
C3—C11	1.4948 (15)	C15—H15	0.9500
C4—C5	1.4110 (15)	C16—C17	1.3864 (18)
C4—H4	0.9500	C16—H16	0.9500
C5—C10	1.4198 (14)	C17—H17	0.9500
C5—C6	1.4212 (15)	C18—H18A	0.9800
C6—C7	1.3607 (17)	C18—H18B	0.9800
C6—H6	0.9500	C18—H18C	0.9800
C7—C8	1.4166 (16)	C19—H19A	0.9800
C7—H7	0.9500	C19—H19B	0.9800
C8—C9	1.3668 (16)	C19—H19C	0.9800
			
C2—O2—C18	116.93 (8)	O1—C11—C3	120.03 (10)
C8—O3—C19	117.45 (9)	C12—C11—C3	119.46 (9)
C2—C1—C10	120.71 (9)	C13—C12—C17	119.52 (11)
C2—C1—H1	119.6	C13—C12—C11	121.46 (10)
C10—C1—H1	119.6	C17—C12—C11	118.97 (10)
O2—C2—C1	124.67 (9)	C14—C13—C12	120.04 (12)
O2—C2—C3	114.85 (9)	C14—C13—H13	120.0
C1—C2—C3	120.42 (10)	C12—C13—H13	120.0
C4—C3—C2	119.11 (10)	C15—C14—C13	120.24 (13)
C4—C3—C11	119.23 (9)	C15—C14—H14	119.9
C2—C3—C11	121.62 (9)	C13—C14—H14	119.9
C3—C4—C5	121.82 (10)	C14—C15—C16	120.00 (12)
C3—C4—H4	119.1	C14—C15—H15	120.0
C5—C4—H4	119.1	C16—C15—H15	120.0
C4—C5—C10	118.63 (10)	C15—C16—C17	119.92 (13)
C4—C5—C6	122.61 (10)	C15—C16—H16	120.0
C10—C5—C6	118.76 (10)	C17—C16—H16	120.0
C7—C6—C5	120.92 (10)	C16—C17—C12	120.23 (12)
C7—C6—H6	119.5	C16—C17—H17	119.9
C5—C6—H6	119.5	C12—C17—H17	119.9
C6—C7—C8	120.02 (10)	O2—C18—H18A	109.5
C6—C7—H7	120.0	O2—C18—H18B	109.5
C8—C7—H7	120.0	H18A—C18—H18B	109.5
O3—C8—C9	125.00 (10)	O2—C18—H18C	109.5
O3—C8—C7	114.12 (10)	H18A—C18—H18C	109.5
C9—C8—C7	120.88 (10)	H18B—C18—H18C	109.5
C8—C9—C10	120.06 (10)	O3—C19—H19A	109.5
C8—C9—H9	120.0	O3—C19—H19B	109.5
C10—C9—H9	120.0	H19A—C19—H19B	109.5
C1—C10—C9	121.37 (10)	O3—C19—H19C	109.5
C1—C10—C5	119.28 (10)	H19A—C19—H19C	109.5
C9—C10—C5	119.34 (10)	H19B—C19—H19C	109.5
O1—C11—C12	120.47 (10)		
			
C18—O2—C2—C1	−8.42 (15)	C8—C9—C10—C1	178.71 (10)
C18—O2—C2—C3	168.77 (9)	C8—C9—C10—C5	−0.15 (15)
C10—C1—C2—O2	176.97 (9)	C4—C5—C10—C1	0.12 (15)
C10—C1—C2—C3	−0.07 (16)	C6—C5—C10—C1	−179.74 (9)
O2—C2—C3—C4	−175.98 (9)	C4—C5—C10—C9	179.01 (9)
C1—C2—C3—C4	1.34 (16)	C6—C5—C10—C9	−0.85 (15)
O2—C2—C3—C11	1.64 (14)	C4—C3—C11—O1	51.76 (14)
C1—C2—C3—C11	178.96 (9)	C2—C3—C11—O1	−125.86 (11)
C2—C3—C4—C5	−1.91 (16)	C4—C3—C11—C12	−126.07 (10)
C11—C3—C4—C5	−179.58 (9)	C2—C3—C11—C12	56.32 (14)
C3—C4—C5—C10	1.18 (16)	O1—C11—C12—C13	−156.48 (11)
C3—C4—C5—C6	−178.96 (10)	C3—C11—C12—C13	21.34 (15)
C4—C5—C6—C7	−179.10 (10)	O1—C11—C12—C17	20.86 (15)
C10—C5—C6—C7	0.75 (16)	C3—C11—C12—C17	−161.33 (10)
C5—C6—C7—C8	0.35 (17)	C17—C12—C13—C14	0.16 (17)
C19—O3—C8—C9	2.94 (16)	C11—C12—C13—C14	177.48 (10)
C19—O3—C8—C7	−176.95 (10)	C12—C13—C14—C15	1.68 (18)
C6—C7—C8—O3	178.50 (10)	C13—C14—C15—C16	−1.88 (19)
C6—C7—C8—C9	−1.40 (17)	C14—C15—C16—C17	0.2 (2)
O3—C8—C9—C10	−178.60 (9)	C15—C16—C17—C12	1.61 (19)
C7—C8—C9—C10	1.28 (16)	C13—C12—C17—C16	−1.80 (17)
C2—C1—C10—C9	−179.52 (9)	C11—C12—C17—C16	−179.19 (11)
C2—C1—C10—C5	−0.65 (15)		

### Hydrogen-bond geometry (Å, °)

**Table:**

*D*—H···*A*	*D*—H	H···*A*	*D*···*A*	*D*—H···*A*
C4—H4···O1^i^	0.95	2.58	3.4439 (13)	151
C18—H18B···O3^ii^	0.98	2.42	3.3742 (15)	164

Symmetry codes: (i) −*x*+2, −*y*, −*z*+1; (ii) *x*−1, *y*, *z*−1.
